# Emulsion PCR (ePCR) as a Tool to Improve the Power of DGGE Analysis for Microbial Population Studies

**DOI:** 10.3390/microorganisms8081099

**Published:** 2020-07-23

**Authors:** Lucilla Iacumin, Francesca Cecchini, Marco Vendrame, Giuseppe Comi

**Affiliations:** Department of Agricultural, Food, Environmental and Animal Science, University of Udine, via Sondrio 2/A, 33100 Udine, Italy; cecchini.francesca79@gmail.com (F.C.); marco.vendrame@uniud.it (M.V.); giuseppe.comi@uniud.it (G.C.)

**Keywords:** emulsion-PCR, DGGE, direct molecular methods, bacterial ecology

## Abstract

To the authors’ knowledge, this is the first report of the use of emulsion-Polymerase chain reaction (e-PCR) coupled with denaturing gradient gel electrophoresis (DGGE) analysis. In the present work the effectiveness of ePCR in improving the power of the DGGE technique for microbial population studies was tested. Our results indicated that ePCR results in uniform amplification of several DNA molecules, overcoming the major limitations of conventional PCR, such as preferential amplification and DNA concentration dependence. Moreover, ePCR-DGGE resulted in higher sensitivity when compared to conventional PCR-DGGE methods used for studying microbial populations in a complex matrix. In fact, compared to conventional PCR, the DGGE profiles of ePCR products permitted the detection of a higher number of the species that were present in the tested sample.

## 1. Introduction

Conventional methods for microbial enumeration, identification, and characterization are insufficient for monitoring specific species in complex, mixed-species microbial communities. Denaturing gradient gel electrophoresis (DGGE) or temperature gradient gel electophoresis (TGGE), introduced by Muyzer, et al. [1], has been widely and successfully used to study microbial communities in food, making it a well-established tool for investigating microbial ecology [2,3,4,5,6,7,8,9,10,11,12]. However, it has some disadvantages, many of which introduced by Polymerase Chain Reaction PCR itself, and it can carry bias. Not all of the present species have similar sensitivities, and the different amounts of the species present in the sample can affect the concentration and detectability of the extracted DNA. Differential or preferential amplification of rDNA genes by PCR was reported by Reysenbach [13] and Suzuki and Giovannoni [14]. When preferential PCR amplification occurs by using extracted metagenomic bacterial DNA from complex communities, certain species may remain undetected by DGGE [2]. More recently, high-throughput sequencing (HTS) technologies have been demonstrated to be a powerful approach to study microbial biodiversity in a wide range of environments, including food. Franciosa et al. [15] reported that mixed amplicons obtained from complex ecosystems can be sequenced and reveal novel microbial fingerprints of so far cryptic populations (identified as operational taxonomic units). Over the last two decades, HTS technologies became ubiquitous in microbial ecology studies, in part due to the progressive reduction of costs. However, the costs for HTS still remain high in comparison to ePCR/DGGE analysis. The costs for HTS may differ across countries or depend on direct access to a sequencing unit, which are expensive and require extensive expertise. In addition, data analysis and verification of sequencing data are a crucial point, which may generate artificial results on the base of imprecise data modulations.

Moreover, HTS technologies, in addition to being subject to the same bias of PCR [16,17], can be affected by some intrinsic limitations: (a) DNA is a recalcitrant molecule, which remains long time after cell death; and (b) only a relative quantification of the genera and/or species is possible. For the above reasons, HTS is limited in its general application. Ultimately, methods are needed which can span from 5 up to 60 days, in particular for when ecological studies are made for the duration of food fermentation processes. Another key point is the detection and identification of species with cell numbers of less than 10^3^ cfu/g [18]. It has been recognized that HTS data, despite providing information at high taxonomic resolution from a wide range of samples, poorly reflect the actual relative abundance of phylotypes, casting doubt on the reliability of data interpretation [19].

Nakano [20] demonstrated that the water in oil (*w*/*o*) emulsion PCR (ePCR) method can solve many biases of traditional PCR, among all the compartmentalization of template DNA molecules reducing the competition between fragments of different lengths and in different concentrations, thus diminishing the bias for amplifying smaller fragments and increasing the concentration of the initial template DNA even if the number of templates is very limited and the segregation of template DNA molecules in each droplet of the emulsion prevents recombination between homologous or partially homologous gene fragments during PCR, thus eliminating the synthesis of short, chimeric products and other artefacts [2]. The principle is to disperse the template DNA molecules in a thermo-stable water-in-oil emulsion at a concentration where, statistically, a few droplets contain more than one gene amplified in situ by PCR to give >10^3^ copies in each droplet [21]. This method is becoming a powerful technique for high-throughput assays in chemistry and biology [22]. In fact, ePCR has been already used to increase the PCR sensitivity in many research fields, in which an absolute quantification of a single target nucleic acid was needed, such as detection of virus, genetically modified organisms, bacterial pathogens, adhesion studies in biofilm structures, and single mutation detection. Its use is often coupled to digital PCR. This last evolution, also called droplet digital PCR (ddPCR), is considered to be the third generation of PCR. After the ePCR reaction, obtained by microfluidics technology, the droplets stream individually pass through the reader for fluorescence analysis. Although this innovative automation of the system allows efficient process management, which reduces the risk of laboratory errors or cross-contamination, it does not currently allow population studies, for which further processing of the generated amplicons are essential [23,24,25,26,27].

In this study, for the first time, ePCR coupled to DGGE analysis was optimized and tested, and the effectiveness in investigating the mixed-species microbial communities using culture-dependent and -independent methods was determined.

## 2. Materials and Methods

### 2.1. Sampling

Three different experiments were performed to compare the capabilities of ePCR and conventional PCR to amplify gene fragments from unique microbial DNA targets in the same sample by using universal 16S primers: (i) using a suspension of lactic acid bacteria (LAB) DNAs extracted from pure cultures, at various concentration ratios; (ii) using DNA extracted from bacterial cells collected in bulk from agar plates after traditional microbiological sampling of fermented sausages; (iii) using DNA directly extracted from sourdough, fermented sausages and food-grade starch (used as an ingredient in food preparations). The experiments were replicated at least 10 times. All positive PCR amplifications were compared by DGGE profiling of individual microbial fingerprints.

### 2.2. DNA Preparation and Extraction 

For the first experiment (i) standard strains of lactic acid bacteria were cultured overnight at 30 °C in De Man, Rogosa e Sharpe broth (MRS, Oxoid, Milan, Italy). A sample (1 mL) of each culture was centrifuged at 14,000× *g* for 10 min at 4 °C, and the pellet was subjected to DNA extraction according to Querol, et al. [28], modified as reported in Iacumin, et al. [29], as follows: 500 μL of 50 mg/mL of Lysozyme from chicken egg white (Sigma-Aldrich, Milan, Italy), 1 M of sorbitol (Sigma-Aldrich, Milan, Italy) and 0.1 M EDTA (Invitrogen Thermo Fisher, Milan, Italy), pH 7.4 were added to the cellular pellet. The tubes were incubated at 37 °C for 2 h in water bath HAAKE SC 150 L (Thermo Scientific, Marietta, OH, USA). The tubes were then centrifuged at 12,500× *g* for 10 min using a Microcentrifuge Model 16K (Bio-Rad, Hercules, CA, USA). After centrifugation, supernatant was discarded. The pellet was resuspended in 500 μL of 50 mM of Tris-HCl (Sigma-Aldrich, Milan, Italy) and 20 mM of EDTA (Invitrogen Thermo Fisher, Marietta, OH, USA) pH 7.4. 50 μL of 10% SDS Solution was added into the tubes. The samples were incubated at 65 °C for 30 min in water bath HAAKE SC 150 L (Thermo Scientific, Marietta, OH, USA). After 30 min, 200 μL of 5 M Potassium Acetate Solution was immediately added into the tubes. The samples were placed in ice for 30 min. Then, the tubes were centrifugated at 14,000× *g* for 5 min. Supernatant containing DNA was transferred to a new sterile tube and 1 mL of ice-cold absolute ethanol (Carlo Erba, Milano, Italy) was added in order to wash the DNA. The tubes were centrifugated at 14,000× *g* for 10 min. Supernatant was eliminated and 500 μL of ice-cold 70% ethanol (Carlo Erba, Milano, Italy) was added for a second wash. The tubes were finally centrifugated at 14,000× *g* for 5 min. The supernatant was removed. The DNA pellet was incubated at 37 °C overnight (15 h) to dry the pellet. After drying, the pellet was resuspended in 50 μL of American Society for Testing and Materials (ASTM) class 1 MilliQ sterile water and 1 μL of RNasi (Sigma-Aldrich, Milan, Italy) was added. Tubes were incubated in water bath HAAKE SC 150 L (Thermo Scientific, Marietta, OH, USA) at 37 °C for 1 h in order to digest co-extracted RNA. DNA was quantified using the Qubit dsDNA assay kit (Thermo Fisher Scientific, Waltham, MA, USA) and then standardized at 50 ng µL^−1^. Two different mixtures of DNA extracted from lactic acid bacteria were prepared for the experiment. In mixture 1 (mix 1) DNA of *Lactobacillus sakei* (DSMZ 6333), *Lactococcus lactis* (DSMZ 20481), *Leuconostoc mesenteroides* (DSMZ 20241) and *Pediococcus pentosaceus* (DSM 20336) were added at the same concentration (100 ng·µL^−1^). In mixture 2 (mix 2), the DNA of the lactic acid bacteria was added at different concentrations: 500 ng·µL^−1^ *Lb. sakei*, 300 ng·µL^−1^ *Lc. lactis*, 150 ng·µL^−1^ *Leuc. mesenteroides*, and 50 ng·µL^−1^ *P. pentosaceus*. One microlitre of each DNA mixture was used for both conventional PCR and ePCR, corresponding to 100 ng for mixture 1 (25 ng per each one of the 4 different species) and 300 ng for mixture 2 (corresponding to 125 ng of DNA from *Lb. sakei*, 75 ng of DNA from *Lc. lactis*, 37.5 ng of DNA from *Leuc. mesenteroides*, and 12.5 ng of DNA from *P. pentosaceus)*.

For the second experiment (ii), a traditional microbiological plate count of coagulase-negative catalase-positive cocci (CNCPC) of samples taken from raw meat sausages during the initial fermentation was set up (Iacumin, et al. [9]). Aliquots (0.1 mL) of serial dilutions of fermented sausage homogenate in 0.25 × Ringer’s solution (1:10) (Oxoid, Milan, Italy) were used to inoculate MSA agar plates (Oxoid, Milan, Italy), then incubated at 30 °C for 48 h under aerobic conditions. After counting colonies, the cells from all individual plates (10^−2^ to 10^−6^) were harvested with a sterile spatula according to Ercolini, et al. [30].

Following harvesting, 1 mL of ASTM class 1 MilliQ sterile water was added to the cells and all solutions were standardized to OD 0.1 at 600 nm. Then, 1 mL of the solutions was centrifuged at 14,000× *g* for 10 min at 4 °C for cell sedimentation, and the pellet was subjected to DNA extraction according to the method previously described. DNA was quantified by using the Qubit dsDNA assay kit (Thermo Fisher Scientific, Waltham, MA, USA) and then standardized to a concentration of 100 ng µL^−1^.

For the third experiment (iii) different food samples were used: fermented sausages [9], sourdough [4], and food-grade starch. Ten (10) g of food samples were homogenized in a stomacher bag with 90 mL of saline-peptone water (8 g·L^−1^ NaCl and 1 g·L^−1^ bacteriological peptone, Oxoid, Milan, Italy) for 3 min. Five (5) mL were then centrifuged at 6000× *g* for 10 min, the pellet was re-suspended in 1 mL Tris-EDTA buffer (TE, 10 mM Tris, 5 mM EDTA, pH 8). Then DNA extraction was performed as described previously. DNA was quantified by using the Qubit dsDNA assay kit (Thermo Fisher Scientific, Waltham, MA, USA) and then standardized to a concentration of 100 ng·µL^−1^.

### 2.3. Conventional PCR Protocol

For the first and second experiments (i, ii), the universal primers P1V1 and P2V1 were used for the amplification of a 180 bp target in the variable V1-region of the 16S rRNA gene as described by Klijn et al. [31]. A GC clamp (5′-CGC CCG CCG CGC CCC GCG CCC GTC CCG CCG CCC CCG CCC G-3′) was added to the forward primer (P1V1) according to Sheffield et al. [32]. The reaction mixture and PCR program of temperature cycling was performed according to Cocolin et al. (2004) [33]. In the second experiment (ii), bovine serum albumin (BSA, 1 mg·mL^−1^, Sigma-Aldrich, Milan, Italy) was added to the PCR reaction mix in the second experiment (ii) and one microliter (1 µL) (100 ng) of DNA extracted was used as template. 

To amplify the DNA directly extracted from foods, the universal primers P1V1/P2V1 were used for sourdough and fermented sausages (as previously described), however, for food-grade starch, the universal primers BA338f-GC (GC-clamp sequence: 5’-CGCCCGCCGCGCGCGGCGGGCGGGGCGGGGGCACGGGGGG-3′) and UN518 [1], which amplify a hypervariable region of 16S rDNA [32], were used to obtain an amplification product of 240 bp. DNA amplifications were performed in a final volume of 25 μL, containing 1 µL (100 ng) of template DNA, 10 mM Tris HCl (pH 8.3), 50 mM KCl, 1.5 mM MgCl_2_, 0.2 mM deoxynucleoside triphosphates (dNTPs), 1.25 U *Taq* polymerase, and 0.2 μM each primer (reagents, Invitrogen, Milan, Italy). A PTC-220 DNA Engine Dyad MJ Research thermal cycler (Celbio, Milan, Italy) was used to conduct the amplification cycle: initial touchdown step in which the annealing temperature was lowered from 65 to 55 °C in intervals of 1 °C every 2 cycles; ten additional cycles were performed with annealing at 55 °C. Denaturation was performed at 95 °C for 1 min, and extension was performed at 72 °C for 1 min 30 s. 

Then, to verify the amplification reaction, amplicons were visualized by electrophoresis in a 0.5× Tris-borate-EDTA (TBE) 1.5% agarose gel (Sigma-Aldrich, Milan, Italy) at 100 V for 45 min and finally subjected to DGGE analysis.

### 2.4. ePCR Protocol

The oil-surfactant mixture was prepared by thoroughly mixing the following components in a 50 mL centrifuge tube at 25 °C: Span 80 (4.5% (*v*/*v*)), Tween 80 (0.4% (*v*/*v*)), Triton X-100 (0.05% (*v*/*v*)) (Sigma-Aldrich, Milan, Italy) and mineral oil to a final volume of 50 mL. 

The oil-surfactant mixture (400 μL) was transferred to a 2 mL CryoTube vial (Cryo.S^TM^, Greiner bio-one Italia srl, Milan, Italy) and stirred at 1000× *g* on a magnetic stirrer using a 3 × 8 mm stir bar. The aqueous phase (PCR mixture) was prepared as previously described in paragraph *PCR protocol*, with a total volume of 260 µL. Two hundred (200) μL of aqueous phase was added to the oil-surfactant mixture in a dropwise manner over a period of 1.5 min and then stirred for 5 min. The emulsion was distributed as 50 μL aliquots into 10 PCR tubes. As a control (not emulsified control; NEC) 50 μL of the aqueous phase were used. Each mixture was subjected to the same temperature-cycling parameters as previously described in paragraph 2.3. The amplified, emulsified PCR reactions were pooled into a 1.5 mL microcentrifuge tube and centrifuged at 13,000× *g* for 5 min at 25 °C to break the *w*/*o* emulsion. The upper (oil) phase was discarded. To remove the remaining oil from the emulsion, 1 mL of water-saturated diethyl ether (Sigma-Aldrich, Milan, Italy) was added to the samples, mixed, and the upper (solvent) phase was then discarded. This washing step was repeated twice. The solvent residue was then removed by vacuum centrifuging for 5 min at 25 °C. To increase the sensitivity of the system, one microliter of the aqueous phase obtained, containing the amplicons, was subjected to a second PCR in a total volume of 50 μL, using the same conditions. All reactions were then checked to verify that amplicons were synthetized by electrophoresis on an agarose gel (1.5%, Sigma-Aldrich, Milan, Italy) in 0.5× Tris-borate-EDTA (TBE, Sigma-Aldrich, Milan, Italy) and finally subjected to DGGE analysis.

All experiments were carried out in triplicate.

### 2.5. DGGE and Bands Identification

DGGE analysis and bands identification was performed according to Iacumin et al. [9], using a denaturing gradient from 30% to 50% (100% corresponds to 7 M urea and 40% (*w*/*v*) formamide) for experiment (i); 40% to 60% for experiment (ii); and 30% to 60%, increasing in the direction of the electrophoretic run, for experiment (iii). Briefly, after gel staining, bands were excised and eluted overnight at 4 °C in sterile water, and, after re-amplification, only the products migrating as one single band and to the same position with respect to the control were amplified as described above using the primer without the GC clamp. Amplification products were then cloned into the pGEM-T Easy vector (Promega, Milan, Italy) following manufacturer’s instructions. Clones were verified as described above (co-migration with control), and the inserts in appropriate clones were sequenced at a commercial facility (Eurofins MWG GmbH, Martinsried, Germany). 

Sequence comparisons were performed using the BLAST program [34]. The choice was set on standard databases nucleotide collection (*nr*/*nt*). Program selection was optimized for highly similar sequences (megablast) and default search parameters were used (max target sequences, 100; automatically adjust parameters for short input sequences, ON; expected threshold, 10; word size, 28; max matches in a query range, 0; match/mismatch, 1, −2; gap Costs, linear; filter, low complexity regions; mask, mask for lookup table only). 

## 3. Results and Discussion

The efficiency of DNA amplification by PCR depends not only on the reaction conditions used but also on the property of the DNA to be amplified. Accessibility to primer hybridization following denaturation is depending on each different DNA template that is present in the mixture, and DNA templates are not be equally accessible, while DNA template length and folding characteristics may influence the PCR efficiency. As Stolovitzky and Cecchi [35] demonstrated, longer molecules will be affected by a decrease in efficiency before the shorter one; the guanine-plus-cytosine (G + C) content conditions the efficiency of PCR, and this is particularly important when PCR is applied to microbial populations, in which genomes vary widely, resulting in preferential amplification of templates with low G + C content [16,17,35,36]. This PCR bias, as well as different binding energies resulting from the use of degenerate primers, is of concern when several DNA templates are amplified simultaneously [13,14,37]. To overcome these problems, a new PCR-based analysis called “emulsion PCR” has been developed. The reaction mixture containing the template DNA is first emulsified into the bulk oil phase and then amplified by PCR. Amplification occurs only in the droplets containing the template DNA and not in droplets without template DNA. After the first step, PCR substrates in the template-containing droplets are exhausted, while those without template DNA still contain PCR substrates. Therefore, to provide the substrates for further amplification, the *w*/*o* emulsion is centrifuged to unite all droplets before carrying out the second step. Although this is a two-step PCR method, it offers a clear advantage over the conventional method by increasing the sensitivity of the analysis [20].

In this study, for the first time, traditional PCR and ePCR coupled with DGGE analysis were compared and evaluated for their effectiveness in amplifying different bacterial species using artificially constituted suspensions, as well as food matrices. 

The study has shown that DGGE fingerprints corresponding to the amplicons obtained by ePCR have more bands than DGGE fingerprints of the amplicons obtained by conventional PCR from the same samples. 

**Figure 1:**
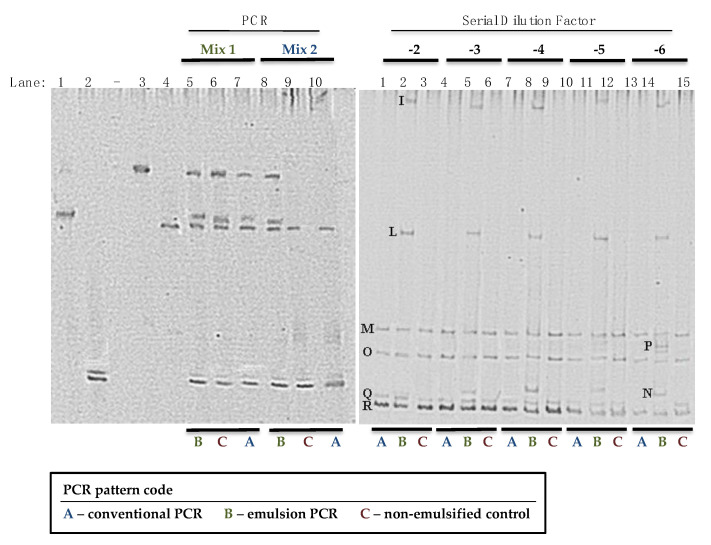
Denaturing gradient gel electrophoresis (DGGE) profiles of mixtures of amplicons obtained from DNA extracted from lactic acid bacteria (A) and DNA extracted directly from bulk-collected coagulase-negative catalase-positive cocci (CNCPC) cells. Panel A: Lane 1, *Lc. lactis*; lane 2, *Leuc. mesenteroides*; lane 3, *P. pentosaceus*; lane 4, *Lb. sakei*; lane 5, ePCR (mix 1); lane 6, not emulsified control (NEC), non-emulsified control (mix 1); lane 7, conventional PCR (mix 1); lane 8, ePCR (mix 2); lane 9, NEC, non-emulsified control (mix 2); lane 10, conventional PCR (mix 2). Mix 1: DNA of *Lactobacillus sakei* (DSMZ 6333), *Lactococcus lactis* (DSMZ 20481), *Leuconostoc mesenteroides* (DSMZ 20241) and *Pediococcus pentosaceus* (DSM 20336) at the same concentration (100 ng·µL^−1^). Mix 2: DNA of the lactic acid bacteria was added at different concentrations: 500 ng·µL^−1^ *Lb. sakei*, 300 ng·µL^−1^ *Lc. lactis*, 150 ng·µL^−1^ *Leuc. mesenteroides* and 50 ng·µL^−1^ *P. pentosaceus*. Panel B: PCR, conventional PCR; ePCR, emulsion PCR; NEC, non-emulsified control; 10^−2^, 10^−3^, 10^−4^, 10^−5^, 10^−6^, sample dilution on MSA agar at which the colonies were harvested in bulk and subjected to total DNA extraction and PCR/ePCR analysis.

In the first part of the study (i), the goal was to verify the capability of e-PCR to overcome the limitations of conventional PCR assays with focus on increasing the efficiency to detect otherwise cryptic bacterial populations in mixed cultures. ePCR and PCR were performed individually on two different mixtures of DNA molecules isolated from standard bacterial strains. In the first case (using mix 1: *Lactobacillus sakei*, *Lactococcus lactis*, *Leuconostoc mesenteroides* and *Pediococcus pentosaceus*, 100 ng·µL^−1^), the fingerprints obtained by conventional PCR and ePCR methods were identical in terms of number and intensity of the bands (Figure 1, panel A, lane 5, 6, and 7, respectively). In contrast, using mix 2 (500 ng·µL^−1^ Lb. *sakei*, 300 ng·µL^−1^ *Lc. lactis*, 150 ng·µL^−1^ *Leuc. mesenteroides* and 50 ng·µL^−1^ *P. pentosaceus*), the bands belonging to all standard bacterial strains were visible only in the DGGE profile of the ePCR-amplified product (Figure 1 (A), lane 8).

In fact, as observed in lanes 9 and 10, the bands corresponding to *P. pentosaceus* and *Lc. lactis* were not visible in the conventional PCR-amplified profile, nor in the not emulsified control (NEC). This result confirmed the increased efficiency of ePCR over conventional PCR, because it was able to amplify microorganisms that were present at low concentrations. 

In part (ii) of the study, the aim was to verify the efficiency of amplification and, consequently, the ability of ePCR to detect different microbial species that were grown on MSA agar plates after serial dilutions of the sample by traditional microbiological sampling. Samples were previously analyzed using traditional microbiological counts and culture-dependent molecular methods [9]. In this experiment, all colonies growing on the surface of each MSA agar plate were collected “in bulk” and subjected to ePCR and PCR followed by DGGE analysis to obtain their fingerprints. This approach has already been used for analyzing dairy and meat products and has provided a “semi-quantitative” characterization of the microbial species present and their concentration dynamics [2,38]. Analysis of DGGE fingerprint patterns and the corresponding dilutions provides information about the dominant species present and allows the determination of the concentration of each species detected in the DGGE profile of the original sample. 

The results obtained clearly demonstrate the presence of four additional bands in the fingerprints obtained after ePCR-DGGE, in comparison to fingerprints obtained after conventional PCR-DGGE (Figure 1, panel B). The identification of each band of the fingerprint was reported in Table 1. 

DNA of *Staphylococcus equorum* (band M), *St. succinus* (band O), and *St. xylosus* (bands Q and R) were present in all samples and at all dilutions. DNA of *St. haemoliticus* (band I), *St. epidermidis* (band L), *St. aureus* (band N), and *St. carnosus* (band P) were detected only in the samples amplified by ePCR. The trial demonstrated that ePCR was more effective in the amplification of the species present in the sample.

Finally, the goal of part (iii) was to evaluate ePCR vs. PCR-DGGE in direct profiling of microbiologically complex food samples using culture-independent methods. In this case as well we used some samples that were previously studied [4,9]. Culture-independent methods are considered a rapid approach for analyzing microbial communities, widely used even in food products [39]. Different food matrices were considered: sourdough, fermented sausage, and food-grade starch.

**Figure 2:**
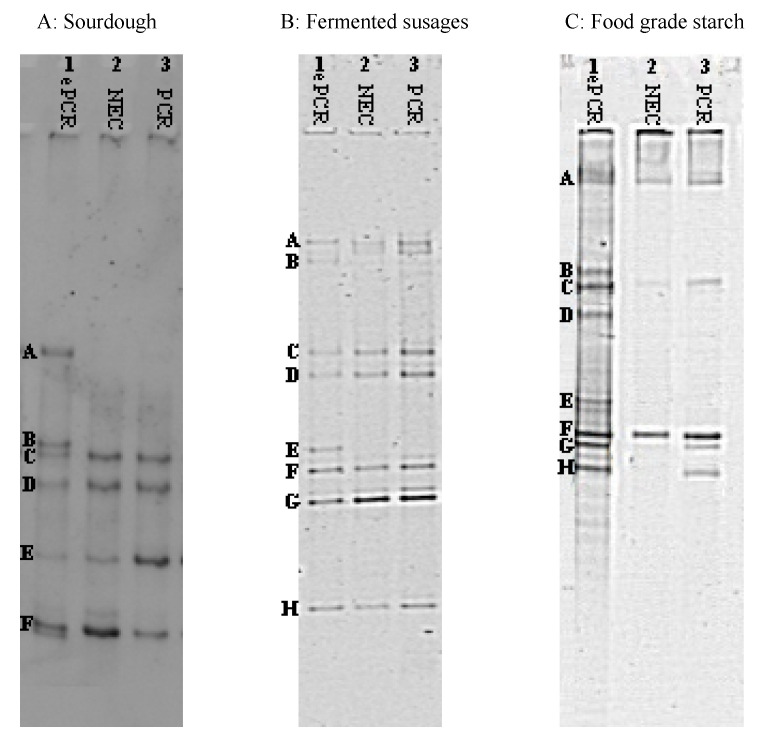
DGGE profiles of PCR amplicons obtained from DNA extracted directly from the food samples. Panel A: sourdough; panel B: fermented sausage; panel C: Food-grade starch; Lane 1, ePCR product; lane 2, non-emulsified PCR product; lane 3, conventional PCR product. Identification of bands is reported in Table 2.

Considering the sourdough sample (Figure 2, panel A), 6 bands were obtained using the ePCR-DGGE versus only 4 bands in the not emulsified control (NEC) and using the PCR-DGGE. The species *Leuconostoc fructosus* (band C), *Pediococcus pentosaceus* (band D), *Lactobacillus paralimentarius* (band E) and *Lactococcus lactis* (band F) were pointed out by both the techniques, but *Lb. brevis* (band A) and *Weissella cibaria* (band B) were solely stressed by ePCR-DGGE (Table 2). This last species was previously never detected in the same samples [4].

As far as the fermented sausage sample is concerned (Figure 2, panel B), the DGGE fingerprints differ for one only band, which correspond to band E (*Staphylococcus equorum*, Table 2). Seven (7) other bands migrated at the same position in the gel (A–H) and highlighted the presence of the species *Lb. sakei*, *Lb. curvatus*, *St. xylosus*, *Lactococcus garviae*, *Lb. sakei* (after sequencing, band A and F led both back to the same species), *P. pentosaceus*, and *Macrococcus caseolyticus*. In agreement with the previous results, in this case as well, *Staphylococcus equorum* was not found by Iacumin et al. [9].

Finally, considering the food-grade starch (Figure 2, panel C), an interesting result was obtained. The use of ePCR was effective in increasing the strength of DGGE analysis in detecting different species in a complex microbial community in food and their ingredients. Compared to DGGE analysis after conventional PCR, three additional bands were detected in the fingerprint obtained by ePCR-DGGE. Upon sequencing of the excised bands (Table 2), *Bacillus pseudofirmus* (band A), B. cereus (bands C and F), *B. thuringiensis* (band G), and *B. subtilis* (band H) were detected in samples subjected to ePCR, non-emulsified PCR, and conventional PCR. However, in the ePCR-DGGE profile, three additional bands corresponding to *Staphylococcus epidermidis* (band B), *B. thuringiensis* (band D), and *B. licheniformis* (band E) were visible. 

In some cases, different bands led to the same identified species (*Bacillus cereus* and *Bacillus thuringiensis*, as well as *Lb. sakei* in Figure 2 Panel B, and S. *xylosus* in Figure 1 and Table 1). This depends on the fact that some strains present DGGE profiles formed by more than one band, at different position of the gel, due to the amplification of multicopies of the 16S rRNA gene, which contained differences detectable by DGGE, as described by Cocolin et al. [40,41].

This last experiment further confirmed the higher efficiency of ePCR. 

This novel ePCR-DGGE method requires no special devices and can be performed using a conventional thermal cycler and a magnetic stirrer, but is more time consuming and requires good manual skills by the professionals. However, due to the preexisting ddPCR, it could be easily automated, solving its limitations. Moreover, considering its effectiveness in solving the most relevant PCR bias, it should be suggested also in combination with HTS technologies to overcome their limitations and obtain a more accurate description of the species that compose complex ecological systems [16,17].

## 4. Conclusions

This is the first report of using ePCR-DGGE analysis. All of the results confirmed the efficiency and utility of ePCR. In fact, all DGGE profiles, corresponding to the amplicons obtained by ePCR, showed more bands than the DGGE profiles of the same amplicons obtained by conventional PCR. This suggests that it is possible to circumvent DNA concentration- and type-dependent preferential amplification by using ePCR. Because DGGE and HTS analysis are still widely used for microbial population studies in food microbiology, it is important to emphasize that the use of ePCR results in a more accurate picture of the microbial species that are present in a particular environment, allowing the limitations of both to be overcome. Our results demonstrate the reliability, sensitivity, and efficiency of ePCR, suggesting that it has great potential for practical applications in microbiological detection, and its application in further studies can give more evidence of the results obtained, in particular for food products or industrial fermentation.

## Figures and Tables

**Table 1:** Identification of the bands obtained by ePCR and PCR-DGGE analysis of the coagulase-negative catalase-positive cocci (CNCPC) bulk-collected samples (Figure 1).

Band(s)	Size	Closest Relative	% Identity	Accession Number
**I**	119	*St. haemoliticus*	99	GQ304781
**L**	120	*St. epidermidis*	100	AM157417
**M**	117	*St. equorum*	98	AY37597
**N**	121	*St. aureus*	100	CP003033
**O**	123	*St. succinus*	99	MK015781
**P**	120	*St. carnosus*	100	AM295250
**Q**	123	*St. xylosus*	99	AB626129
**R**	124	*St. xylosus*	99	AB626129

**Table 2:** Identification of the bands obtained by ePCR and PCR-DGGE of the starch sample.

Sample	Band(s) ^a^	Size	Closet Relative	% Identity	Accession Number ^b^
Panel A Sourdough					
	A	218	*Lb. brevis*	100	AB362618
	B	223	*Weissella cibaria*	100	DQ885576
	C	223	*Leuc. fructosus*	100	AF360737
	D	209	*Ped. pentosaceus*	100	AB362605
	E	199	*Lb. paralimentarius*	99	AB289235
	F	203	*Lc. lactis*	100	EF694031
Panel B Fermented Sausages					
	A		*Lb. sakei*	99	AB682465
	B		*Lb. curvatus*	99	JN382074
	C		*St. xylosus*	99	AY126259
	D		*Lc. garviae*	100	FJ611799
	E		*St. equorum*	98	AY37597
	F		*Lb. sakei*	99	AB124845
	G		*Ped. pentosaceus*	97	AB681648
	H		*Macr. caseolyticus*	98	AY126157
Panel C Food-Grade Starch					
	A	155	*B. pseudofirmus*	98	JN566125
	B	165	*St. epidermidis*	100	HM161755
	C	180	*B. cereus*	100	JQ178332
	D	171	*B. thuringiensis*	100	JN411477
	E	130	*B. licheniformis*	100	JQ388689
	F	158	*B. cereus*	98	HQ290100
	G	160	*B. thuringiensis*	98	JN411477
	H	157	*B. subtilis*	98	JN408331

^a^ Bands are numbered as indicated on the DGGE gels shown in Figure 2. ^b^ Accession number of the sequence of the closest relative found by BLAST search.
